# Imperfect Synthetic Controls

**DOI:** 10.1002/jae.70035

**Published:** 2026-01-14

**Authors:** David Powell

**Affiliations:** University of Pennsylvania, Philadelphia, Pennsylvania, USA

**Keywords:** convex hull, parallel trends, synthetic control estimation, transitory shocks, C23, C13, C21

## Abstract

The synthetic control method assumes the existence of a perfect synthetic control, which cannot exist if the outcomes are functions of transitory shocks with nonzero asymptotic variance and may not exist even in expectation for the treated unit. This paper first shows the benefits of estimating synthetic controls for all units. If the treated unit composes part of the synthetic control for any untreated unit, the treatment effect is independently identified by the synthetic outcome minus the outcome of the untreated unit in the post-treatment period (divided by the synthetic control weight on the treated unit outcome). This paper introduces an estimator which generates synthetic controls for all units and develops moment conditions which are valid given transitory shocks. I also introduce a weighting metric which asymptotically excludes units without appropriate synthetic controls. The paper exploits the estimator’s construction of multiple estimates of the treatment effect to produce valid inference even when the number of control units is small. The estimator is used to evaluate the repeal of Wisconsin’s handgun purchase waiting period on suicide rates.

## 1 | Introduction

Empirical research often relies on panel data to compare outcome changes in a unit adopting a policy of interest to units that did not adopt the policy. While difference-in-differences methods are often used, the synthetic control method (SCM)—developed in Abadie and Gardeazabal (2003), Abadie et al. (2010), and Abadie et al. (2015)—offers researchers an alternative approach. The synthetic control estimator uses a data-driven method to create a weighted average of the other units to act as a counterfactual for the treated unit and has been called “[a]rguably the most important innovation in the evaluation literature in the last fifteen years” (Athey and Imbens 2017).

The SCM relies on the assumption that there exists a weighted average of the pre-intervention outcomes of the control units equal to the pre-intervention outcomes of the treated unit, where the weights are nonnegative and sum to one. Let $Y_{it}$ represent the outcome for unit i in time period t. The SCM “convex hull” assumption is that $\sum_{j=2}^{N}w_{j}^{1}Y_{jt}=Y_{1t}$ for all pre-interventions periods, where $w_{j}^{1}$ represents the weight on unit j to construct the counterfactual for unit 1, the treated unit. This paper addresses concerns with this assumption.

The first concern is that, even in expectation, the treated unit’s pre-intervention outcomes may not be within the convex hull of the control units’ outcomes. To address this problem, this paper recognizes that the treated unit may be part of an appropriate synthetic control for one or more untreated units. The post-treatment outcome differences for these units are informative of the policy effect. This insight relaxes the SCM assumption such that only one of the following conditions must hold: (a) $\sum_{j=2}^{N}w_{j}^{1}Y_{jt}=Y_{1t}$ or (b) $\sum_{j\neq i}^{N}w_{j}^{i}Y_{jt}=Y_{it}$ with $w_{1}^{i}>0$ for some i. The latter condition permits identification of the policy effect even when the treated unit does not have an appropriate synthetic control. Given that we never know whether the convex hull assumption holds for the treated unit or not, the estimator in this paper constructs synthetic controls for *all* units and estimates the policy effect using the differences in the outcomes and the differences in the policy variable for all units. This approach provides an opportunity to place more weight on units with better synthetic control fits.

It is also well-recognized that $\sum_{j=2}^{N}w_{j}^{1}Y_{jt}=Y_{1t}$ is unlikely to hold in practice for all time periods (see page 24 of Abadie (2021)). As the pre-period grows, this condition cannot hold given independent (across units) transitory shocks, inducing bias (Ferman and Pinto 2021). Asymptotically, this bias may converge to zero if the variance of the transitory shocks converges to zero, which is also a restrictive assumption (see Section 3.2.1). Otherwise, the bias remains even for large pre-periods. Many recent modifications to the SCM also impose the $\sum_{j=2}^{N}w_{j}^{1}Y_{jt}=Y_{1t}$ assumption but relax the restrictions on the weights. However, this assumption is problematic given transitory shocks, regardless of the flexibility permitted for the weights.

The proposed approach in this paper introduces moment conditions which address this issue, producing asymptotically unbiased estimates even when $\sum_{j\neq i}^{N}w_{j}^{i}Y_{jt}\neq Y_{it}$ for all i and regardless of the size of the transitory shocks. Instead, this paper relies on assumptions of the form $\sum_{j\neq i}^{N}w_{j}^{i}E\left[Y_{jt}\right]=E\left[Y_{it}\right]$, alleviating the problems induced from transitory shocks and only requiring the convex hull assumption to hold in expectation. The cost of this modification is that every unit i either (1) must have an appropriate synthetic control, as defined by $\sum_{j\neq i}^{N}w_{j}^{i}E\left[Y_{jt}\right]=E\left[Y_{it}\right]$ or (2) be part of one or more appropriate synthetic controls, as defined by $\sum_{j\neq k}^{N}w_{j}^{k}E\left[Y_{jt}\right]=E\left[Y_{kt}\right]$ with $w_{i}^{k}>0$.

This paper intersects with a growing literature evaluating the properties of the SCM and proposing modifications. Doudchenko and Imbens (2016) permit negative weights and weights that do not sum to one to permit inference in cases in which the convex hull assumption does not hold, a practice also adopted in Amjad et al. (2018). Ferman and Pinto (2021) show that a “demeaned” synthetic control approach may have less bias. Similarly, Doudchenko and Imbens (2016) suggest including unit fixed effects to account for fixed differences across units.^1^

To address concerns with transitory shocks, the literature has typically relied on postestimation bias reduction methods or pre-processing approaches. These approaches often implicitly unravel the restrictions on the weights and require extrapolation. Ben-Michael et al. (2021a) add a postestimation bias reduction method, while Amjad et al. (2018) propose a pre-processing denoising method to account for transitory shocks under the assumption that these shocks are independent across units and time. Arkhangelsky et al. (2021) show that using both unit- and time-specific synthetic control weights in a two-way fixed effects regression operates as a bias reduction method.^2^

Most related to this paper, Fry (2024) introduces a GMM estimation technique in which units are stratified into control units and a disjoint set of units whose outcomes are used as instruments. The instruments address concerns about transitory shocks, similar to the benefits of instrumental variables in the context of measurement error. This approach has several advantages, though it does require a large set of untreated units such that some can act as instruments. Shi et al. (2021) consider a similar GMM approach where the instruments are outcomes from other units and/or covariates.

The proposed approach in this paper maintains the SCM’s restrictions on the weights, preserving the conceptual advantages of the original method. Alternative approaches require out-of-sample extrapolations by relaxing the restrictions on the weights or use postestimation debiasing methods which do not enforce those restrictions.^3^ By maintaining the restrictions on the weights, the introduced method avoids out-of-sample extrapolations and does so without requiring the inclusion and estimation of unit fixed effects or demeaning the outcome by unit. In addition, the proposed method uses simple moment conditions which permit transitory shocks and imperfect fits between units and their synthetic controls without additional pre-estimation or postestimation processing. Given the implausibility of assuming no transitory shocks (or even sufficiently small transitory shocks), it is important to address this issue.

The proposed approach directly addresses the nonexistence of perfect synthetic controls. For this reason, I refer to the estimator of this paper as the imperfect synthetic control method (ISCM). The lack of perfect synthetic controls is the norm in empirical applications so the proposed method should have broad applicability.

The first insight that synthetic controls for untreated units can identify the treatment effect unambiguously relaxes the traditional “convex hull” assumption. That contribution by itself can be used with SCM (or modified synthetic control approaches) to estimate the treatment effects of interest. However, ISCM leverages this insight further to directly address the implausibility of the traditional $\sum_{j=2}^{N}w_{j}^{1}Y_{jt}=Y_{1t}$ assumption. ISCM replaces this assumption with a convex hull assumption that must only hold in expectation. However, the ISCM estimator requires its convex hull assumption to hold for at least one unit and, most likely, multiple units. In addition, I introduce a weighting metric which asymptotically (as the number of pre-intervention time periods grows) excludes units without an appropriate synthetic control from impacting the main estimate. With SCM, it is common to decide postestimation whether a synthetic control is an appropriate counterfactual, typically by visual inspection, though metrics have also been considered in the literature (Hollingsworth and Wing 2020; Parast et al. 2020). The proposed weighting metric removes the decision of which units to use from the researcher and produces a treatment effect using only units with appropriate synthetic controls.

Finally, I propose an inference procedure which leverages ISCM’s construction of counterfactuals for multiple units. By doing so, ISCM (potentially) produces multiples estimates of the treatment effect. The proposed inference procedure uses the mean and variance of these estimates to construct a t-statistic, following “finite inference” methods introduced in Ibragimov and Müller (2010) and Canay et al. (2017). An advantage of this approach is that it produces informative p-values even when the “donor pool” is small. A common problem in applications using SCM with the traditional permutation test is that it may not be possible to generate enough counterfactuals to provide p-values meeting standard statistical thresholds. This occurs in the application of this paper, which has eight states in the donor pool. Using a permutation test, the smallest possible p-value in such a case would be greater than 0.10.

**FIGURE 1 |:**
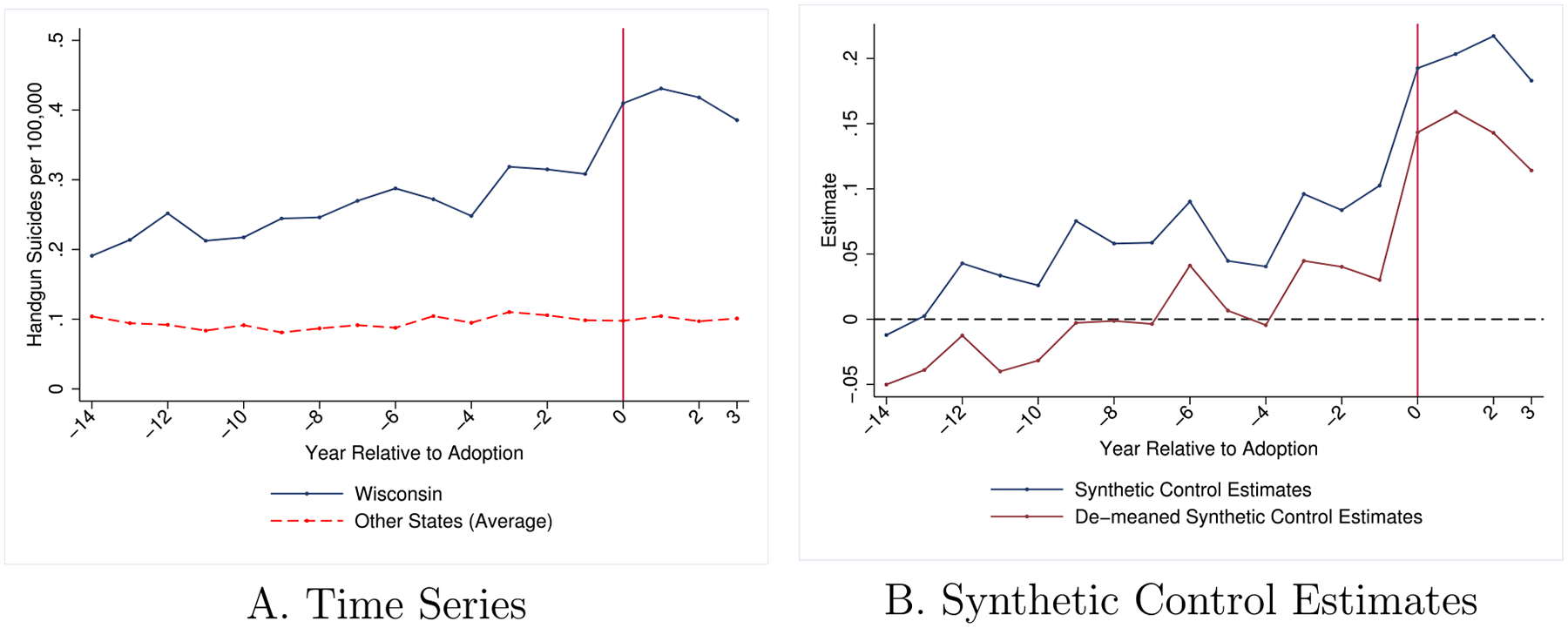
Handgun suicide rates. *Source:* NVSS data. The outcome is handgun suicide deaths per 100,000. The data are monthly and all analyses are performed at the monthly level. The “other states” in Panel A refer to the 8 states which also had waiting periods during this time period. These 8 states are the donor pool for the synthetic control estimates in Panel B. The results are aggregated to the annual level for presentation purposes. Panel A shows the raw time series. Panel B provides the SCM and demeaned SCM results.

I apply ISCM to evaluate the consequences of the June 2015 repeal of Wisconsin’s 48-h handgun purchase waiting period on suicide rates. Waiting periods for the purchase of handguns are intended to prevent impulsive acts that might end in a homicide or suicide. For an analysis of Wisconsin’s repeal, there are eight states which had waiting periods during the same time period and did not repeal them (Oliphant 2022; Edwards et al. 2018; Cherney et al. 2018).^4^ These states serve as potential contributors to the construction of a counterfactual for Wisconsin’s handgun suicide rate. Compared to those eight states, Wisconsin’s handgun suicide rate was trending upward prior to the repeal (shown in Figure 1A), casting doubt on the appropriateness of traditional difference-in-differences methods. In addition, Wisconsin had the highest rate of handgun suicide rates for 73 of the 161 months in the preperiod used in the analysis, clearly violating the “convex hull” assumption of the SCM, even in expectation. Results from the traditional SCM are provided in Figure 1B and show that the preperiod Wisconsin rates are not well-approximated by the counterfactual produced by the SCM.

In the next section, I include more background about the SCM and recent modifications in the literature. I introduce the estimator in steps. Section 3 discusses limitations of the SCM and how the proposed modifications address these restrictions. Section 4 introduces the ISCM estimator and discusses its properties. Section 5 discusses the inference. Section 6 provides results for the impact of Wisconsin’s 48-h handgun purchase waiting period repeal. Section 7 concludes the study.

## 2 | Background

### 2.1 | The SCM

I assume N units and T time periods. Unit 1 is exposed to the treatment in periods $T_{0}+1$ to T and unexposed in periods 1 to $T_{0}$. All other N-1 units are unexposed in all time periods. I assume large $T_{0}$ throughout this paper. Outcomes are defined by the following:

(1) $$Y_{it}=\alpha_{it}D_{it}+L_{it}+\epsilon_{it},$$

(2) $$\text{where}\;D_{it}=\left\{\begin{array}{ll} 1 & \text{if}\;i=1\;\text{and}\;t>T_{0},\\ 0 & \text{otherwise}. \end{array}\right.$$

$L_{it}$ is the (unobserved) “systematic component” and following Arkhangelsky et al. (2021) and Athey et al. (2021), I consider $L_{it}$ fixed throughout this paper. $D_{it}$ represents the treatment variable, while $\epsilon_{it}$ represents the transitory component, where $E\left[\epsilon_{it}|D_{it}\right]=0$ such that $E\left[Y_{it}|D_{it}=0\right]=L_{it}$. In a latent factor model, $L_{it}=\lambda_{t}\mu_{i}$, where $\lambda_{t}$ is a 1×F vector of common unobserved factors and $\mu_{i}$ is an F×1 vector of factor loadings.

For the main discussion of ISCM, I preserve the setup above while extensions are considered in Section 4.4. The goal of traditional synthetic control estimation is to find a weighted combination of units (which exists by assumption) such that

(3) $$\sum_{j=2}^{N}w_{j}^{1}L_{jt}=L_{1t}\;\text{for all}\;t,$$

where $w_{j}^{1}$ represents the (true) weight on unit j to construct the synthetic control representing unit 1 (see equation 4 of Abadie et al. (2010) for the factor model version of this assumption). For the SCM, it is unnecessary to designate that the weight is *for* the construction of the synthetic control for unit 1, but this notation will be useful below. If this condition holds, then $\sum_{j=2}^{N}w_{j}^{1}Y_{jt}$ serves as a counterfactual for the treated unit. With latent factor models, this assumption is equivalent to $\sum_{j=2}^{N}w_{j}^{1}\mu_{j}=\mu_{1}$. Since the $L_{it}$ terms are unobserved, the SCM and many modifications of the SCM instead assume the condition $\sum_{j=2}^{N}w_{j}^{1}Y_{jt}=Y_{1t}$. The implications of this condition not holding with equality are discussed in Section 3.2.1.

Let $w_{1}={\left(w_{2}^{1},\ldots ,w_{N}^{1}\right)}^{\prime }$ such that $w_{j}^{1}\geq 0$ for all 2≤j≤N and $\sum_{j=2}^{N}w_{j}^{1}=1$. The candidate weights are represented by $\phi_{1}={\left(\phi_{2}^{1},\ldots ,\phi_{N}^{1}\right)}^{\prime }$. The weights are estimated using

$${\overset{ˆ}{w}}_{1}=\underset{\phi_{1}}{\text{argmin}}\sum_{t=1}^{T_{0}}{\left(Y_{1t}-\sum_{j=2}^{N}\phi_{j}^{1}Y_{jt}\right)}^{2}\;\text{s.t.}\;\phi_{j}^{1}\geq 0\;\text{for all}\;2\leq j\leq N\;\text{and}\;\sum_{j=2}^{N}\phi_{j}^{1}=1.$$

The synthetic control estimator involves a constrained optimization to find the weighted average of the donor states which is “closest” to the treated unit in terms of pretreated outcomes.^5^ The treatment effect estimate for period $t>T_{0}$ is

(4) $${\overset{ˆ}{\alpha }}_{1t}=Y_{1t}-\sum_{j=2}^{N}{\overset{ˆ}{w}}_{j}^{1}Y_{jt}.$$

### 2.2 | Modified Synthetic Control Approaches

An emerging literature addresses how to modify the SCM when the convex hull assumption does not hold. Doudchenko and Imbens (2016) relax the convex hull assumption by (1) permitting negative weights, (2) allowing the sum of the weights to not equal one, and (3) permitting permanent additive differences across units. These modifications extrapolate to predict counterfactual outcomes. Other modifications in the literature also implement regularization/penalization approaches instead of strictly imposing the SCM restrictions on the weights (e.g., Amjad et al. (2018); Ben-Michael et al. (2021a)).

The third modification of Doudchenko and Imbens (2016) extends synthetic control estimation to cases in which the convex hull assumption does not hold solely because of level differences across units. Similarly, Ferman and Pinto (2021) recommend demeaning the outcomes by unit. These approaches address cases in which the treated unit has the largest or smallest outcomes in pretreatment periods; however, the convex hull assumption may not hold (even in expectation) after adjusting for such level differences. If an additive term is the only reason that the convex hull assumption does not hold, then ISCM should address this problem without explicitly including an additive unit term.

Other work uses the traditional SCM and then pairs it with postestimation bias reduction methods. Ben-Michael et al. (2021a) propose a bias correction due to inexact matching, equivalent to permitting negative weights. Arkhangelsky et al. (2021) propose “synthetic difference in differences” (SDID) as a double bias reduction method, assuming large N and T. Other approaches use preprocessing methods (Amjad et al. 2018; Xu 2017) to extract underlying components of the outcomes before using the SCM.

ISCM preserves many of the features of the original SCM such as enforcing the restrictions on the weights and the convex hull assumption—avoiding extrapolation—while not requiring separate unit fixed effects. It also does not require any preprocessing or postestimation bias reduction methods, which often implicitly require extrapolation.

## 3 | Model

The modified estimator will require synthetic controls for all units. The vector of weights to construct a synthetic control for unit i is represented by $w_{i}=\left(w_{1}^{i},\ldots ,w_{i-1}^{i},w_{i+1}^{i},\ldots ,w_{N}^{i}\right)$, where $w_{j}^{i}$ represents the weight given to unit j for the creation of the synthetic control for unit i. The weights are constrained as before. I define the set of possible weighting vectors to create the unit i synthetic control by

(5) $${𝒲}_{i}=\left\{w_{i}|\;|||\;|\sum_{j\neq i}w_{j}^{i}=1,w_{j}^{i}\geq 0\;\text{for all}\;j\right\}.$$

In this section, I introduce the two modifications of this paper. First, I relax the convex hull assumption by permitting the treated unit to be outside the convex hull of the other units. Second, I show how jointly estimating synthetic controls for all units can permit transitory shocks, represented by $\epsilon_{it}$ in Equation (1), to the outcome variable without inducing (asymptotic) bias. I initially consider these modifications independently, followed by a more formal discussion in Section 4.

### 3.1 | Treated Unit Outcomes Outside of the Convex Hull

The SCM assumes that the pretreated outcomes of the treated unit are within the “convex hull” of the outcomes of the other units. In this section, I assume that the weights are known to the researcher.

#### 3.1.1 | Synthetic Controls for all Units

The approach of this paper estimates synthetic controls for all units. This idea offers the opportunity to use an alternative assumption to Equation (3):^6^

(6) $$\sum_{j\neq i}^{N}w_{j}^{i}L_{jt}=L_{it}\;\text{for all}\;t,\;\text{for some}\;i\;\text{with}\;w_{1}^{i}>0.$$

This condition states that the “convex hull assumption” holds for unit i>1, and the treated unit is part of the synthetic control for unit i>1. Under this assumption, for $t>T_{0}$ and i>1,

$$Y_{it}-\sum_{j\neq i}^{N}w_{j}^{i}Y_{jt}=\left(L_{it}-\sum_{j\neq i}^{N}w_{j}^{i}L_{jt}\right)+\left(\alpha_{it}D_{it}-\sum_{j\neq i}^{N}w_{j}^{i}\alpha_{jt}D_{jt}\right)+\left(\epsilon_{it}-\sum_{j\neq i}^{N}w_{j}^{i}\epsilon_{jt}\right),\;=-\alpha_{1t}w_{1}^{i}+\left(\epsilon_{it}-\sum_{j\neq i}^{N}w_{j}^{i}\epsilon_{jt}\right).$$

The first term on the right-hand side, $L_{it}-\sum_{j\neq i}^{N}w_{j}^{i}L_{jt}$, is equal to zero by assumption (Equation (6)). Since $D_{it}=0$ for all i>1, the second term reduces to $-\alpha_{1t}w_{1}^{i}D_{1t}=-\alpha_{1t}w_{1}^{i}$ for $t>T_{0}$. Assuming that $w_{1}^{i}>0$ and that $D_{1t}$ is independent of $\epsilon_{it}$ for all i, then $\alpha_{1t}$ is identified by the above equation. Thus, even in cases in which there is no appropriate synthetic control for unit 1, unit 1 may receive a positive weight as part of the synthetic control for another unit, identifying the treatment effect.

Equation (6) relaxes the traditional synthetic control assumption since only one of the assumptions (Equation (3) or Equation (6)) is required to hold. The proposed approach is *not* equivalent to permitting negative weights or weights summing to more than one to construct a synthetic control for unit 1. The condition expressed in Equation (6) is constrained by Equation (5), producing “in-sample” predictions for unit i in the same manner as the traditional SCM demands in-sample predictions for unit 1. Any set of weights satisfying Equation (6) condition can be rewritten as a set of weights on units i>1 to construct a counterfactual for unit 1. These weights will be greater than or equal to one or less than or equal to zero. For $t\leq T_{0}$, a simple rearranging produces $Y_{1t}=\frac{1}{w_{1}^{i}}Y_{it}-\sum_{j\neq 1,i}\frac{w_{j}^{i}}{w_{1}^{i}}Y_{jt}$, where $\frac{1}{w_{1}^{i}}\geq 1$ and $-\frac{w_{j}^{i}}{w_{1}^{i}}\leq 0$. The proposed approach, however, is not permitting any set of weights to construct a counterfactual for unit 1. Instead, they are constrained by the usual conditions to construct an appropriate synthetic control for unit i. ISCM relies on units in which it is possible to construct in-sample predictions.

#### 3.1.2 | Estimating Treatment Effects

The proposed estimation procedure involves creating synthetic controls for all units and regressing $\left(Y_{it}-\sum_{j\neq i}{\overset{ˆ}{w}}_{j}^{i}Y_{jt}\right)$ on $\left(D_{it}-\sum_{j\neq i}{\overset{ˆ}{w}}_{j}^{i}D_{jt}\right)$. Researchers can use the preperiod synthetic control fit to determine which units are appropriately within the convex hull of the other units and include these units in this regression. This approach is equivalent to the typical method employed with the SCM of deciding whether or not the pretreatment fit is adequate. In Section 3.3, I introduce a weighting metric which will systematize this method, eliminating the need for the researcher to make such judgments. However, this traditional approach of evaluating the preperiod visually or using other metrics is still available. Define the following variable:

(7) $$a_{i}=\left\{\begin{array}{l} {\overset{‾}{a}}_{i}>0\;\text{if}\;\sum_{j\neq i}w_{j}^{i}L_{jt}=L_{it}\;\text{for all}\;t,\\ 0\;\text{otherwise.} \end{array}\right.$$

For now, I assume that the appropriateness of the synthetic control—that is, the value of $a_{i}$—is known to the researcher. The empirical analog of $a_{i}$, denoted $a_{i}(\overset{ˆ}{w})$, will be defined later. The intuition is that there exists a weight which is equal to 0 when the convex hull assumption does not hold. In all other cases, the weight is positive. In practice, some units will have higher weights than others and contribute more to the estimation of the treatment effect. The method of this paper is to construct synthetic controls for all units and then estimate the relationship between the difference in outcomes and difference in the policy variable using weighted least squares (WLS):

(8) $${\overset{ˆ}{\alpha }}_{1t}=\underset{\alpha }{\text{argmin}}\frac{1}{2N}\sum_{i=1}^{N}a_{i}\left(\overset{ˆ}{w}\right){\left[\left(Y_{it}-\sum_{j\neq i}{\overset{ˆ}{w}}_{j}^{i}Y_{jt}\right)-\alpha \left(D_{it}-\sum_{j\neq i}{\overset{ˆ}{w}}_{j}^{i}D_{jt}\right)\right]}^{2}.$$

A benefit of using all units is that it is possible (in principle or, as discussed below, asymptotically) to estimate the treatment effect using only units with valid synthetic controls. Even when the treated unit does not have an appropriate synthetic control, the treatment effect can still be identified.

The alternative to the proposed approach is using only the treated unit even in cases in which the relevant assumptions fail. The insight of this section stands alone as an independent contribution, providing an opportunity to estimate treatment effects even when the traditional SCM or modified SCMs fail. The rest of the paper uses this insight further to address bias due to transitory shocks.

### 3.2 | Synthetic Controls Given Transitory Shocks

Abadie et al. (2010) discuss the usefulness of weights such that $\mu_{1}=\sum_{j=2}^{N}w_{j}^{1}\mu_{j}$ (i.e., $L_{it}=\sum_{j=2}^{N}w_{j}^{1}L_{jt}$ for all t), but this relationship must be estimated using pre-intervention outcomes. The outcomes, however, are noisy measures of the variables of interest, and this noise can prevent consistent estimation of the weights (Ferman and Pinto 2021).

#### 3.2.1 | Bias in SCM

The SCM moment conditions have the form, for k>1,

(9) $$\frac{1}{T_{0}}\sum_{t=1}^{T_{0}}Y_{kt}\left(Y_{1t}-\sum_{j=2}^{N}w_{j}^{1}Y_{jt}\right)=\frac{1}{T_{0}}\sum_{t=1}^{T_{0}}Y_{kt}\left(\epsilon_{1t}-\sum_{j=2}^{N}w_{j}^{1}\epsilon_{jt}\right),$$

where the equality follows from Equation (1) and a convex hull assumption. This expression is generally not equal to zero in expectation given the mechanical correlation between $Y_{kt}$ and $\epsilon_{kt}$, though it could converge to 0 if $\frac{1}{T_{0}}\sum_{t}\epsilon_{kt}^{2}\overset{p}{\to }0$, a restrictive assumption. Otherwise, this problem is not alleviated as $T_{0}$ increases (Ferman and Pinto 2021).^7^

#### 3.2.2 | Modified Moment Conditions

I propose a modification of the above moment condition (and continue to assume that the synthetic control weights and $a_{i}$ weights are known):

(10) $$\frac{1}{T_{0}}\sum_{t=1}^{T_{0}}Y_{kt}\left(Y_{1t}-\sum_{j\neq 1,k}^{N}w_{j}^{1}Y_{jt}-w_{k}^{1}{\overset{ˆ}{Y}}_{kt}\right),$$

where ${\overset{ˆ}{Y}}_{kt}$ is the estimate from a WLS regression:

(11) $${\overset{ˆ}{Y}}_{kt}=\frac{\sum_{i\neq k}a_{i}\left[\left(Y_{it}-\sum_{j\neq i,k}w_{j}^{i}Y_{jt}\right)w_{k}^{i}\right]+a_{k}\sum_{j\neq k}w_{j}^{k}Y_{jt}}{\sum_{i\neq k}a_{i}{\left(w_{k}^{i}\right)}^{2}+a_{k}\times 1}\;=L_{kt}+\frac{\sum_{i\neq k}a_{i}\left[\left(\epsilon_{it}-\sum_{j\neq i,k}w_{j}^{i}\epsilon_{jt}\right)w_{k}^{i}\right]+a_{k}\sum_{j\neq k}w_{j}^{k}\epsilon_{jt}}{\sum_{i\neq k}a_{i}{\left(w_{k}^{i}\right)}^{2}+a_{k}}.$$

The expected value of the last term above is equal to zero, meaning $E\left[{\overset{ˆ}{Y}}_{kt}\right]=E\left[Y_{kt}\right]=L_{kt}$. The right-hand side of Equation (11) represents WLS with outcome and explanatory variable defined in the following manner:

$$\text{For all}\;i\neq k,\;\underset{“\text{outcome}”}{\underbrace{Y_{it}-\sum_{j\neq i,k}w_{j}^{i}Y_{jt}}}=w_{k}^{i}\times \underset{“\text{unknown}”}{\underbrace{Y_{kt}}}+\left(\epsilon_{it}-\sum_{j\neq i}w_{j}^{k}\epsilon_{jt}\right),$$

$$\text{For}\;i=k,\underset{“\text{outcome}”}{\underbrace{\sum_{j\neq k}w_{j}^{k}Y_{jt}}}=1\times \underset{“\text{unknown}”}{\underbrace{Y_{kt}}}-\left(\epsilon_{kt}-\sum_{j\neq k}w_{j}^{k}\epsilon_{jt}\right).$$

I designate $Y_{kt}$ as “unknown” in the above equations because it is the parameter of interest for this prediction. Given that it will be necessary to construct predictions for all units, the insight in Section 3.1 becomes further useful. One possible way to estimate predicted values for unit i is to jointly construct a synthetic control for unit i. However, it may be that unit i does not have an appropriate synthetic control. In such cases, it is still possible to use the synthetic controls constructed for other units to estimate predicted values for unit i in the same manner as discussed previously. Equation (11) uses this insight.

In the previous section, I discussed the merits of estimating synthetic controls for every unit, but the synthetic control weights $w_{i}$ were separately estimated from the synthetic control weights $w_{j}$. In this section, I propose estimating all weights jointly.^8^

#### 3.2.3 | Validity of Moment Conditions

Returning to the moment conditions and assuming $L_{it}=\sum_{j\neq i}w_{j}^{i}L_{jt}$ for all t, then

$$E\left[\frac{1}{T_{0}}\sum_{t=1}^{T_{0}}Y_{kt}\left(Y_{it}-\sum_{j\neq i,k}w_{j}^{i}Y_{jt}-w_{k}^{i}{\overset{ˆ}{Y}}_{kt}\right)\right]=E\left[\frac{1}{T_{0}}\sum_{t=1}^{T_{0}}Y_{kt}\left(\epsilon_{it}-\sum_{j\neq i,k}w_{j}^{i}\epsilon_{jt}\right.\left.-w_{k}^{i}\frac{\sum_{m\neq k}a_{m}\left[\left(\epsilon_{mt}-\sum_{j\neq m,k}w_{j}^{m}\epsilon_{jt}\right)w_{k}^{m}\right]+a_{k}\sum_{j\neq k}w_{j}^{k}\epsilon_{jt}}{\sum_{m\neq k}a_{m}{\left(w_{k}^{m}\right)}^{2}+a_{k}\times 1}\right].\right.$$

This equals zero as $\epsilon_{kt}$ never appears in the term in parentheses. If the convex hull assumption does not hold for unit i, then this moment condition will not be equal to zero. However, the $a_{i}$ weights will set it to zero.

#### 3.2.4 | Final Conditions

ISCM estimates all synthetic control weights jointly. Define a set of candidate synthetic control weights $\phi \equiv \left(\phi_{1},\ldots ,\phi_{N}\right)$. The N-1 conditions related to unit i are as follows:

(12) $$M_{i}(\phi )\equiv \left[\begin{array}{c} \frac{1}{T_{0}}\sum_{t=1}^{T_{0}}\left(Y_{it}-\sum_{j\neq i,1}\phi_{j}^{i}Y_{jt}-\phi_{1}^{i}{\overset{ˆ}{Y}}_{1t}(\phi )\right)Y_{1t}\\ \frac{1}{T_{0}}\sum_{t=1}^{T_{0}}\left(Y_{it}-\sum_{j\neq i,2}\phi_{j}^{i}Y_{jt}-\phi_{2}^{i}{\overset{ˆ}{Y}}_{2t}(\phi )\right)Y_{2t}\\ \vdots \\ \frac{1}{T_{0}}\sum_{t=1}^{T_{0}}\left(Y_{it}-\sum_{j\neq i,i-1}\phi_{j}^{i}Y_{jt}-\phi_{i-1}^{i}{\overset{ˆ}{Y}}_{i-1,t}(\phi )\right)Y_{i-1,t}\\ \frac{1}{T_{0}}\sum_{t=1}^{T_{0}}\left(Y_{it}-\sum_{j\neq i,i+1}\phi_{j}^{i}Y_{jt}-\phi_{i+1}^{i}{\overset{ˆ}{Y}}_{i+1,t}(\phi )\right)Y_{i+1,t}\\ \vdots \\ \frac{1}{T_{0}}\sum_{t=1}^{T_{0}}\left(Y_{it}-\sum_{j\neq i,N}\phi_{j}^{i}Y_{jt}-\phi_{N}^{i}{\overset{ˆ}{Y}}_{Nt}(\phi )\right)Y_{Nt} \end{array}\right],$$

where

(13) $${\overset{ˆ}{Y}}_{kt}(\phi )=\frac{\sum_{i\neq k}a_{i}\left[\left(Y_{it}-\sum_{j\neq i,k}\phi_{j}^{i}Y_{jt}\right)\phi_{k}^{i}\right]+a_{k}\sum_{j\neq k}\phi_{j}^{k}Y_{jt}}{\sum_{i\neq k}a_{i}{\left(\phi_{k}^{i}\right)}^{2}+a_{k}\times 1}.$$

Each condition in Equation (12) replaces $Y_{kt}$ with its predicted value when it is enforcing orthogonality with $Y_{kt}$.

### 3.3 | Weighting Metric and Final Moment Conditions

I have assumed that it is known when the convex hull assumption holds for unit i and when it does not. In this section, I define the $a_{i}$ weights. These weights have the advantageous property that, asymptotically (as $T_{0}\to \infty$), they exclude units in which the convex hull assumption does not hold. The weights can be written as

(14) $$a_{i}\left(w\right)\equiv \frac{{\left[M_{i}(w{)}^{\prime }M_{i}(w)\right]}^{-1}}{{\left[\underset{j}{\text{min}}\;M_{j}(w{)}^{\prime }M_{j}(w)\right]}^{-1}}.$$

The benefit of these $a_{i}(w)$ weights is that if unit i does not have an appropriate synthetic control, such that $L_{it}\neq \sum_{j\neq i}w_{j}^{i}L_{jt}$, then $a_{i}(w)\overset{p}{\to }0$ since the denominator tends to infinity.^9^ This property (asymptotically) eliminates units without a valid synthetic control. For units in which $L_{it}=\sum_{j\neq i}w_{j}^{i}L_{jt}$ for all $t,\;a_{i}(w)$ converges to some nonnegative constant $0\leq {\overset{‾}{a}}_{i}\leq 1$. For simplicity, I will assume ${\overset{‾}{a}}_{i}>0$, which means that $M_{i}(w{)}^{\prime }M_{i}(w)$ does not converge to 0 more slowly than the minimum.^10^

The usefulness of these weights is that they make it unnecessary to have prior knowledge concerning which units that the convex hull assumption is valid for. It is also unnecessary to make postestimation decisions about when the convex hull assumption appears to hold. It is typical to estimate synthetic control weights and then determine the appropriateness of the counterfactual using the preperiod fit. This option is still available to the researcher, but these weights systemize the decisions.

In practice, the $a_{i}$ weights place more weight on units with better fits and less weight on units with bad synthetic control fits. The $a_{i}$ weights are also used to construct predicted values during the estimation process. The same logic applies, and these weights serve to, asymptotically, only use units with appropriate synthetic controls when constructing the predicted values.

However, the $a_{i}$ weights need to be evaluated for given synthetic control weights and, therefore, are defined recursively. Let ${\overset{ˆ}{w}}^{\left(s\right)}$ represent the weights generated from iteration s. The estimator uses $a_{i}\equiv a_{i}\left({\overset{ˆ}{w}}^{\left(s\right)}\right)$ in Equation (13) to help generate the predicted ${\overset{ˆ}{Y}}_{it}(\phi )$ values for iteration s+1. Estimation resembles continuously updated generalized method of moments. The final moment conditions are as follows:

$$M\left(\phi \right)\equiv \left(\begin{array}{c} a_{1}\left(\phi \right)M_{1}\left(\phi \right)\\ \vdots \\ a_{N}\left(\phi \right)M_{N}\left(\phi \right) \end{array}\right).$$

The benefit of constructing the moments in this manner is that minimization requires ϕ values such that, for each $i,\;M_{i}(\phi )\overset{p}{\to }0$ or $a_{i}(\phi )\overset{p}{\to }0$. When unit i has an appropriate synthetic control, then $M_{i}(w)\overset{p}{\to }0$; when unit i does not have an appropriate synthetic control, then $a_{i}(w)\overset{p}{\to }0$.

### 3.4 | Estimation

There are N(N-1) moment conditions and N(N-1) synthetic control weights. In principle, for each unit in which $a_{i}=0$, the weighting metric drops N-1 conditions *and* N-1 parameters to estimate such that identification is not impacted. The final optimization uses

$${\left({\overset{ˆ}{w}}_{1},\ldots ,{\overset{ˆ}{w}}_{N}\right)}^{\left(s+1\right)}=\underset{\phi_{1}\in {𝒲}_{1},\ldots ,\phi_{N}\in {𝒲}_{N}}{\text{argmin}}M(\phi {)}^{\prime }M(\phi )$$

with $a_{i}\equiv a_{i}\left({\overset{ˆ}{w}}^{\left(s\right)}\right)$ in Equation (13).

For optimization, I use a Nelder–Mead technique. Optimization is completed when the mean squared difference in the ${\overset{ˆ}{Y}}_{it}$ estimates for all $\left(i,t\leq T_{0}\right)$ between iteration s-1 and s is below a set tolerance level.^11^ I label the total number of iterations necessary S. I summarize the following suggested steps to implement ISCM:

1. Using the traditional SCM, get initial values of the weights for all units $\left(\tilde{w}\right)$. These are the starting values for the optimization.
2. Construct initial “fit” measures $a_{i}(\tilde{w})\equiv \frac{{\left[M_{i}(\tilde{w}{)}^{\prime }M_{i}(\tilde{w})\right]}^{-1}}{{\left[{\text{min}}_{j}M_{j}(\tilde{w}{)}^{\prime }M_{j}(\tilde{w})\right]}^{-1}}$.
3. Construct initial ${\overset{ˆ}{Y}}_{it}(\tilde{w})$ predictions.
4. Select synthetic control weights to minimize $M(\phi {)}^{\prime }M(\phi )$ using the Nelder-Mead optimization technique. Update the ${\overset{ˆ}{Y}}_{it}\left({\overset{ˆ}{w}}^{\left(s\right)}\right)$ predictions continuously using Equation (13).
5. Repeat step 4 until the mean squared change in ${\overset{ˆ}{Y}}_{it}$ over all $\left(i,t\leq T_{0}\right)$ between consecutive iterations is below a specified tolerance level.
6. Estimate the treatment effect using Equation (8).

The treatment effect is estimated separately in the final step. It is not jointly estimated with the w weights. It is recommended to repeat steps 2–6 above with different starting values.

## 4 | ISCM

In this section, I discuss the ISCM more formally. Define $D_{i}\equiv {\left(D_{i1},\ldots ,D_{iT}\right)}^{\prime },\;L_{i}\equiv {\left(L_{i1},\ldots ,L_{iT}\right)}^{\prime }$, and $L\equiv \left(L_{1},\ldots ,L_{N}\right)$. I continue to assume fixed N and large $T_{0}$.

### 4.1 | Conditions

ISCM relies on the following assumptions:

**A1** (Outcomes): $Y_{it}=\alpha_{it}D_{it}+L_{it}+\epsilon_{it}$, where $D_{it}$ is specified in Equation (2), $Y_{it}$ is continuous, $L_{it}$ is fixed, and $\left\|Y_{it}Y_{jt}\right\|<\infty$ for all (it,jt).

**A2** (Existence of Synthetic Controls): For all i, (a) there exists $w_{i}\in {𝒲}_{i}$ such that $L_{it}=\sum_{k\neq i}w_{k}^{i}L_{kt}$ for all t or (b) there exists $w_{j}\in {𝒲}_{j}$ with $w_{i}^{j}>0$ such that $L_{jt}=\sum_{k\neq j}w_{k}^{j}L_{kt}$ for all t.

**A3** (Independence): (a) $E\left[\epsilon_{it}|D_{i},L_{i}\right]=0$; (b) $E\left[\epsilon_{it}\epsilon_{jt}|D_{i}\right.,\;\left.L_{i},D_{j},L_{j}\right]=0$ for all i≠j.

**A4** (Within-Unit Dependence): (a) For each $i,\;\epsilon_{it}$ is a strongly mixing sequence in t of size $-\frac{r}{r-1},\;r>1$; (b) $E{\left|\epsilon_{it}\right|}^{r+\delta }<\infty$ for any δ>0.

**A5** (Weighting): If **A2**(a) holds for unit i, then $a_{i}(w)\overset{p}{\to }{\overset{‾}{a}}_{i}>0$.

### 4.2 | Discussion

Assumptions **A1–A3** generalize conditions presented in previous sections. **A1** nests latent factor models $\left(L_{it}=\lambda_{t}\mu_{i}\right)$, additive forms typically assumed with difference-in-differences $\left(L_{it}=\alpha_{i}+\gamma_{t}\right)$, and other models. Condition **A2** replaces the traditional SCM assumption $Y_{1t}=\sum_{k\neq 1}w_{k}^{1}Y_{kt}$, given the implausibility of the SCM assumption, replacing it is a major motivation of this paper. Notably, it does not require the existence of a synthetic control for the treated unit, and it only requires this condition to hold in expectation.

While Equation (6) unambiguously relaxed the traditional synthetic control assumption (Equation (3)) since only one of the conditions was required to hold, **A2** includes a condition for every unit. This is the cost of avoiding the $Y_{1t}=\sum_{k\neq 1}w_{k}^{1}Y_{kt}$ restriction. In principle, assumption **A2** can be relaxed further. When this assumption does not hold for a unit, then that unit can be excluded from the analysis (though this requires an active decision from the researcher) and the treatment effect itself is still identified if the conditions above hold without that unit.

**A2** does *not* imply that every unit or even most units must have an appropriate synthetic control. To understand the implications of **A2**, note that it may only require one unit to have an appropriate synthetic control. Imagine that the unweighted average of all other units is an appropriate synthetic control for unit i (i.e., $w_{j}^{i}=\frac{1}{N-1}$ for all j≠i), similar to a typical difference-in-differences assumption. In such a case, **A2** is satisfied by that one unit (though there are likely benefits to having additional units with appropriate synthetic controls). More generally, **A2** is satisfied if there exists unit j such that $L_{jt}=\sum_{k\neq j}w_{k}^{j}L_{kt}$ where $w_{k}^{j}>0$ for all k≠j. **A2** permits other combinations of units to have proper synthetic controls—the requirement is simply that every unit either has an appropriate synthetic control or is part of one.

Under these conditions, the synthetic control weights are not necessarily unique as different weights may create the same predicted counterfactuals. Instead, the predicted counterfactuals are unique. Assumption **A4** governs conditions on $\epsilon_{it}$. **A5** is a convenient regularity condition which rules out units with proper synthetic controls receiving a weight of zero (asymptotically). In practice, this would not be problematic.^12^

### 4.3 | Properties

There is only one treated unit so I show that ${\overset{ˆ}{\alpha }}_{1t}$ is asymptotically unbiased as $T_{0}\to \infty$. This property is formalized below and in the Supporting Information Appendix.

**Theorem 4.1.** (Asymptotically Unbiased). *Assume that* **A1–A5** *hold. Then*, ${\overset{ˆ}{\alpha }}_{1t}\overset{p}{\to }\alpha_{1t}+V_{t}$, *where* $E\left[V_{t}\right]=0$ *as* $T_{0}\to \infty$.

See Supporting Information Appendix for details. Given a long postperiod (i.e., large $T-T_{0}+1$) or multiple treated units (see Section 4.4.3), an aggregate estimate may converge to the true average (under additional regularity conditions).

### 4.4 | Extensions

#### 4.4.1 | Measuring Fit and Evaluating Assumptions

It is typical when using the SCM to examine the fit of the synthetic control in the pre-intervention period to evaluate the appropriateness of the convex hull assumption. An equivalent test can be implemented with the proposed method by estimating $\alpha_{1t}$ for each $t\leq T_{0}$ using Equation (8) and plotting each time period’s estimate. This acts as a test of condition **A2**. This approach is similar to an event study approach which estimates the “effects” before the intervention to test for preexisting trends. In this case, the approach also tests for preexisting level differences.

#### 4.4.2 | Multiple Treated Time Periods

It is straightforward to alter Equation (8) to estimate aggregated effects, such as one effect for the full postintervention period:

(15) $${\overset{ˆ}{\alpha }}_{1}=\underset{\alpha }{\text{argmin}}\frac{1}{2N\left(T-T_{0}\right)}\sum_{t=T_{0}+1}^{T}\sum_{i=1}^{N}a_{i}\left(\overset{ˆ}{w}\right){\left[\left(Y_{it}-\sum_{j\neq i}{\overset{ˆ}{w}}_{j}^{i}Y_{jt}\right)-\alpha \left(D_{it}-\sum_{j\neq i}{\overset{ˆ}{w}}_{j}^{i}D_{jt}\right)\right]}^{2}.$$

#### 4.4.3 | Multiple Treated Units

I have assumed that there is only one treated unit, but it is common to study policies adopted by many units. It is straightforward to extend ISCM to these empirical applications since ISCM already requires creating synthetic controls for every unit. If we assume that multiple units adopted the policy of interest at the same time, then implementation of ISCM is identical to the case in which only one unit adopts the policy.

The same approach can be used given differential timing of policy adoption, but the pre-intervention period cannot contain any treated observations. One possibility is to use the time period that is untreated for all units as a common pre-intervention period. An alternative is to exclude early adopters as part of the donor pool for late adopters of the policy.^13^

#### 4.4.4 | Adding Covariates

The specification in Abadie et al. (2010), using a latent factor model framework, includes additional covariates $\left(Z_{it}\right)$ such that $Y_{it}=\alpha_{it}D_{it}+Z_{it}^{\prime }\Pi +\lambda_{t}\mu_{i}+\epsilon_{it}$.^14^ Adding conditions related to the covariates permits estimation of weights which also account for cross-unit variation in the covariates. However, the traditional approach assumes that there exists weights such that there is also a “perfect fit” for the treated unit’s covariate values. This assumption can be restrictive.

Following the approach introduced in Powell (2022), it is possible to jointly estimate the relationship between the covariates and the outcome while also estimating the synthetic control weights by including moment conditions such as the following to those in Equation (12):

$$\frac{1}{T_{0}}\sum_{t=1}^{T_{0}}Z_{it}\left(Y_{it}-Z_{it}^{\prime }\gamma -\sum_{j\neq i}\phi_{j}^{i}\left(Y_{jt}-Z_{jt}^{\prime }\gamma \right)\right).$$

The construction of the counterfactual requires adjusting for the treated observations’ values of the covariates using the estimated coefficients on those variables.

## 5 | Inference

Abadie and Gardeazabal (2003) and Abadie et al. (2010) recommend a permutation test in which a treatment effect is estimated for each unit in the donor pool. Under the null hypothesis of no treatment effect, these placebo tests generate a distribution of estimates which would be observed randomly. The placement of $\overset{ˆ}{\alpha }$ (or a related test statistic) in this distribution generates a p-value under the assumption that the error term is identically distributed across units.^15^

One shortcoming of a permutation test is that it requires enough units such that standard statistical thresholds can potentially be met. ISCM produces multiple estimates of the same treatment effect, permitting the use of “finite inference” methods such as those introduced in Ibragimov and Müller (2010) and Canay et al. (2017). Ibragimov and Müller (2010) show that the mean of the estimates scaled by the standard deviation provides a useful t-statistic that can be used for conservative inference (under certain conditions). Canay et al. (2017) suggest perturbing the estimates around the null hypothesis with Rademacher weights to simulate the distribution of the test statistic.

Let 𝒞 represent the set of units in which $\left(D_{iT}-\sum_{j\neq i}{\overset{ˆ}{w}}_{j}^{i}D_{jT}\right)\neq 0$. Define weights attributed to each unit as

$$v_{i}=\frac{a_{i}(\overset{ˆ}{w}){\left(D_{iT}-\sum_{j\neq i}{\overset{ˆ}{w}}_{j}^{i}D_{jT}\right)}^{2}}{\sum_{i=1}^{N}\left[a_{i}(\overset{ˆ}{w}){\left(D_{iT}-\sum_{j\neq i}{\overset{ˆ}{w}}_{j}^{i}D_{jT}\right)}^{2}\right]},$$

where the denominator normalizes the weights so that the sum is equal to 1. Note that $v_{i}=0$ if i∉𝒞. A regression of $\left(Y_{it}-\sum_{j\neq i}{\overset{ˆ}{w}}_{j}^{i}Y_{jt}\right)$ on $\left(D_{it}-\sum_{j\neq i}{\overset{ˆ}{w}}_{j}^{i}D_{jt}\right)$, weighted by $a_{i}(\overset{ˆ}{w})$, is equivalent to a weighted average of ${\overset{ˆ}{\alpha }}_{i}\equiv \frac{\left(Y_{it}-\sum_{j\neq i}{\overset{ˆ}{w}}_{j}^{i}Y_{jt}\right)}{\left(D_{it}-\sum_{j\neq i}{\overset{ˆ}{w}}_{j}^{i}D_{jt}\right)}$ with the $v_{i}$ weights. Despite indexing ${\overset{ˆ}{\alpha }}_{i}$ by i, each ${\overset{ˆ}{\alpha }}_{i}$ represents an estimate of the treatment effect for unit 1. With $\overset{ˆ}{\alpha }$ defined in Equation (8), the test statistic for null hypothesis $H_{0}:\alpha =\alpha_{0}$ is

(16) $$T=\frac{{\left(\sum_{i\in 𝒞}v_{i}{\overset{ˆ}{\alpha }}_{i}-\alpha_{0}\right)}^{2}}{\frac{N}{N-1}\sum_{i\in 𝒞}v_{i}{\left({\overset{ˆ}{\alpha }}_{i}-\overset{ˆ}{\alpha }\right)}^{2}}.$$

The distribution of T can be simulated by perturbing each $\left({\overset{ˆ}{\alpha }}_{i}-\alpha_{0}\right)$ by drawing randomly from the Rademacher distribution: equal to 1 with probability $\frac{1}{2}$ and equal to −1 with probability $\frac{1}{2}$. The placement of T in the distribution of $T^{\left(k\right)}$ defines a p-value. Reject $H_{0}$ at level b if $T>T^{\left(k\right)}[1-b]$.

The Canay et al. (2017) method requires breaking ties randomly, an approach that would probably not be used in practice. In this context, when the number of units in which $w_{1}^{j}>0$ is small, it may remain impossible to achieve p-values meeting standard statistical thresholds. In such contexts, the Ibragimov and Müller (2010) approach may be valid, though it provides conservative inference. I apply this approach for the application below.

## 6 | Application: Handgun Waiting Periods and Suicide Mortality

Suicide is the tenth leading cause of death in the United States (Hedegaard and Warner 2021), and there is significant policy and research interest in understanding policies which can curb the rising suicide rate (Marcotte and Hansen 2024; Marcotte and Zejcirovic 2020; Powell 2023). Based on a review of the literature, Smucker et al. (2020) conclude that there is “moderate evidence” that waiting periods reduce the rate of firearm suicides.^16^

In June 2015, Wisconsin repealed its 48-h handgun purchase waiting period. Dunton et al. (2022) study time series changes in the suicide rate in Wisconsin after the repeal. Oliphant (2022) implemented a demeaned synthetic control estimator using eight donor states which had waiting periods and did not repeal them.

To analyze the suicide impacts of Wisconsin’s repeal, I use restricted mortality data from the National Vital Statistics System (NVSS) with geographic identifiers for 2002–2019.^17^ ICD-10 code X72 denotes handgun suicides. I use monthly data starting in 2002 such that the preperiod is January 2002 to May 2015. I scale the number of suicides per month by the state population using Census data. The donor pool includes California, Hawaii, Illinois, Iowa, Maryland, Minnesota, New Jersey, and Rhode Island. These states had waiting periods at the start of the analysis period, like Wisconsin, and did not subsequently repeal or change them. Since the repeal occurred June 25, 2015, I drop June 2015 from the analysis. To reduce noise while also providing rich temporal information, I present results in 12-month aggregates based on time relative to June 2015.

I provide traditional and demeaned SCM results in Figure 1B (referenced earlier) for handgun suicide rates. The convex hull assumption is clearly violated, even in expectation, for the SCM. Demeaning the outcome visually centers the preperiod estimates, but this approach still produces biased estimates and does not ensure that the convex hull assumption is met.

I next implement the ISCM estimator. Estimation follows the approach in Section 3.4. I first use the traditional SCM to estimate initial synthetic control weights and construct the $a_{i}$ weights. I then minimize the objective function while regularly updating the $a_{i}$ weights. This is repeated until the mean squared difference in the predicted outcomes between iterations is sufficiently small. The inference procedure in Section 5 is used to generate p-values.

**FIGURE 2 |:**
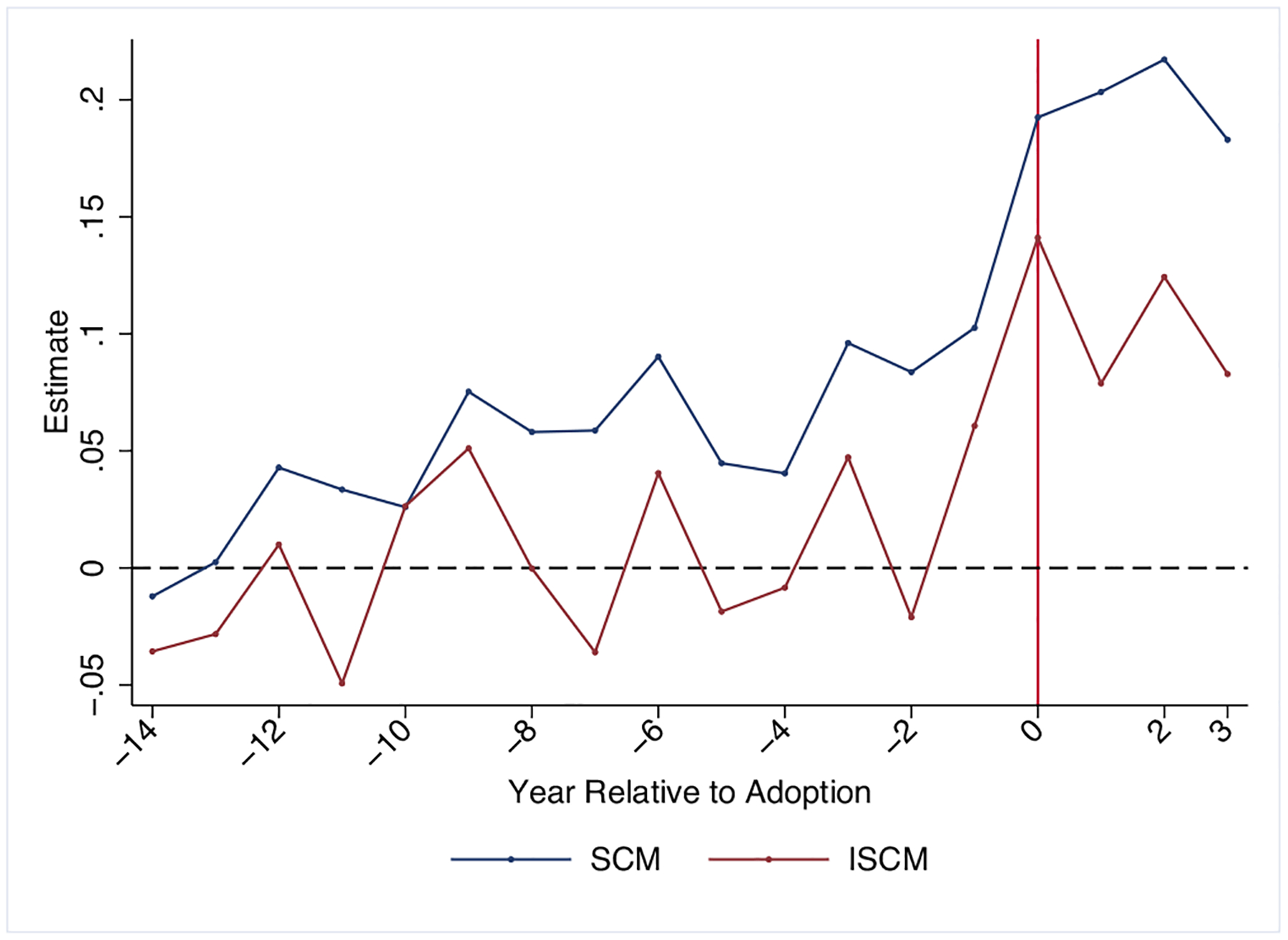
Imperfect synthetic control results. *Source:* NVSS data. The outcome is handgun suicide deaths per 100,000. The data are monthly, and all analyses are performed at the monthly level. The results are aggregated to the annual level for presentation purposes.

The results are provided in Figure 2. Unlike the estimates generated from the SCM and the demeaned SCM (discussed above in Figure 1B), there is less evidence of an upward trend throughout the preperiod. Also, notably, despite not explicitly including additive unit effects or demeaning the outcome, the preperiod estimates hover around zero. At the time of the repeal, we observe an increase in handgun suicide mortality.

The average treatment effect is the average of the 4 years after repeal (Years 0–3 in the figures) and reported in Table 1. I estimate that the repeal increased handgun suicide mortality by 0.11 deaths per 100,000 residents, equivalent to about a 30% increase relative to the outcome prior to repeal. The p-value is 0.046 using the Ibragimov and Müller (2010) inference approach. The main estimate is a weighted average of the state-specific treatment effects for the Wisconsin repeal. Table 1 reports the state-specific estimates along with the percentage of the main effect which that estimate contributed. These are the $v_{i}$ weights and reflect the extent to which each $\alpha_{i}$ is weighted. Iowa and its synthetic counterfactual provide 35.7% of the average effect estimates, while Indiana and its synthetic control provide 34.5%. The average effect is also composed of 19.4% of Mississippi minus its synthetic control while California and Rhode Island receive very small weights (< 1%).^18^ Wisconsin’s treatment effect composes only 9.9% of the average effect. While Wisconsin’s synthetic control is a poor fit, it still receives some weight given that the treated unit has the highest value of ${\left(D_{it}-\sum_{j\neq i}{\overset{ˆ}{w}}_{j}^{i}D_{jt}\right)}^{2}$.

## 7 | Conclusion

The ISCM introduced in this paper addresses the convex hull assumption of the SCM. It does so while avoiding extrapolation, explicitly including additive fixed effects, or using pre-estimation or postestimation adjustment methods. It maintains the original restrictions on the weights and the conceptual advantages of these restrictions. The ISCM relies on the insight that the treated unit may be part of the synthetic control of one or more control units, and the postadoption outcome differences in these units also provide evidence of policy effects. The ISCM develops synthetic controls for all units and then aggregates this information to estimate the policy effect. Even when the convex hull assumption holds for the treated unit, there are potential gains in using more units.

This first insight of this paper unambiguously relaxes the traditional SCM assumptions, providing opportunities to leverage the benefits of synthetic control estimation (SCM or modified approaches) in otherwise inappropriate contexts. Moreover, synthetic control weights are biased since they are estimated to minimize the distance between outcomes, and these outcomes are noisy measures of the underlying trends and shocks of interest. ISCM relies on a set of moment conditions robust to transitory shocks. The idea is to estimate synthetic controls for all units jointly, permitting the construction of predicted values for all units and time periods. The construction of these predicted values is necessary to relax the SCM assumption that $\sum_{j=2}^{N}w_{j}^{1}Y_{jt}=Y_{1t}$. The predicted values are orthogonal to the transitory shocks that are inducing the bias in SCM. The gains of replacing an assumption that does not permit transitory shocks with assumptions that do should be significant given that such shocks are the norm in empirical applications.

This paper also introduces a weighting metric used in the estimation of the synthetic control weights and the treatment effect which, asymptotically, selects the units with appropriate synthetic controls, removing this decision from the researcher. For inference, the paper leverages ISCM’s production of multiple estimates of the same treatment effect. This new approach has benefits over the traditional permutation method even when using the SCM.

The proposed estimation technique is used to study the implications of Wisconsin’s handgun purchase waiting period repeal on handgun suicide rates. The ISCM estimator produces pretreatment estimates centered around zero with little evidence of systematic trends. After repeal, there is visual evidence of an increase in handgun suicide rates. The mean increase over the full postperiod is meaningful and statistically significant from zero. Given the widespread use of synthetic control estimation across many fields, the proposed ISCM approach should have wide applicability.

## Supplementary Material

Appendix

Additional supporting information can be found online in the Supporting Information section. jae70035-0001-supinfo_imperfect synthetic controls-pages-2.pdf

## Figures and Tables

**TABLE 1 |:** Main estimate and state-specific estimates.

	Main estimate	Wisconsin	California	Indiana	Iowa	Mississippi	Rhode Island
Estimate	0.105	0.232	0.336	0.063	0.038	0.232	−1.931
p-value	[0.046]						
Weight		9.94%	0.43%	34.46%	35.73%	19.43%	0.02%

*Note:* This table provides the estimate of the treatment effect generated for each state in which the demeaned treatment variable is nonzero. The p-value for the main estimate is provided in brackets using the proposed Ibragimov and Müller (2010) approach. The “Weight” row shows what share of the total estimate that each state estimate contributes. These are *not* the synthetic control weights but reflect the $v_{i}$ weights discussed in Section 5.

## Data Availability

The data used in this article are drawn from a restricted version of the National Vital Statistics System mortality files. The replication package (https://journaldata.zbw.eu/dataset/imperfect-synthetic-controls) explains how to access these data and includes the code.

## Footnotes

1 Arkhangelsky et al. (2021) account for unit fixed effects after estimating the synthetic control weights.

2 Hollingsworth and Wing (2020) discuss the gains of merging synthetic control estimation with lasso to use a large set of predictors to construct counterfactual outcomes.

3 Kellogg et al. (2021) discuss how the SCM’s avoidance of extrapolation bias can induce interpolation bias and propose combining synthetic control and matching techniques. This paper builds on the SCM and preserves the key features of the method.

4 Some states modified their waiting periods during the time period and were excluded from the analysis.

5 The SCM uses a two-step process in which some variables included in the minimization are weighted more than others if they are better predictors of the pre-intervention outcomes. Since I use the pre-intervention outcomes themselves in this discussion, this weighting matrix is unnecessary (Kaul et al. 2022).

6 In a latent factor framework, this assumption can be written as $\sum_{j\neq i}^{N}w_{j}^{i}\mu_{j}=\mu_{i}$ for some i with $w_{1}^{i}>0$.

7 However, it may be alleviated as N increases if the weights attached to each unit converge to 0 (Ferman 2021).

8 This approach has some parallels with solutions found in the errors-in-variables literature in which a predicted value is used. However, note that an “instrumental variable”-type approach would use ${\overset{ˆ}{Y}}_{kt}\left(Y_{1t}-\sum_{j\neq 1}^{N}w_{j}^{1}Y_{jt}\right)$, which is invalid since ${\overset{ˆ}{Y}}_{kt}$ is correlated with $\epsilon_{jt}$. Fry (2024) introduces an instrumental variable version which uses outcomes in other units as instruments. This estimator must properly categorize units as controls or as instruments.

9 This assumes that at least one unit has an appropriate synthetic control.

10 This assumption is a convenient regularity condition. Alternatively, I could permit ${\overset{‾}{a}}_{i}=0$ for some of these units, but these units could then not be used to relax the convex hull assumption in Equation (6).

11 Alternatively, I could use the mean squared difference in the w estimates. As noted later, the weights themselves are not necessarily unique and may be prone to bounce around while holding the predict counterfactuals stable.

12 Alternatively, I could modify **A2** to account for units with proper synthetic controls which are asymptotically excluded by the weighting metric.

13 Ben-Michael et al. (2021b) discuss synthetic control estimation given staggered adoption.

14 Arkhangelsky et al. (2021) estimate synthetic control weights based only on pretreatment outcomes. These weights are then used in a fixed effects regression which includes time-varying covariates.

15 Alternate approaches and test statistics have also been mentioned in the literature (see Firpo and Possebom (2018), Hahn and Shi (2017), Chernozhukov et al. (2021), and Cattaneo et al. (2019)).

16 Smucker et al. (2020) refer to Ludwig and Cook (2000), Luca et al. (2017), and Edwards et al. (2018).

17 To display the results, I construct 12-month periods relative to the adoption date so the analysis ends in May 2019.

18 All states not listed received a weight of 0.
