# Supplementary material for: Imperfect Synthetic Controls
Source: J Appl Econ (Chichester Engl). Author manuscript; Available in PMC 2026 Jan 28. (PMC12843250; doi:10.1002/jae.70035)
Supplement: Appendix [file NIHMS2138288-supplement-Appendix.pdf]

Online Appendix

Theorem 4.1 (Asymptotically Unbiased)**.** *Assume **A1**-**A5** hold. Then,* $\widehat{\alpha}_{1t} \xrightarrow{p} \alpha_{1t} + V_t$, *where* $E[V_t] = 0$ *as* $T_0 \to \infty$.

Proof.

Part 1: First, assume that there are known a_i weights for equation (13) which meet the conditions of equation (7). Then, for units in which **A2**(a) holds, by Section 3.2.3,

$$E\left[\frac{1}{T_0}\sum_{t=1}^{T_0} Y_{kt}\left(Y_{it} - \sum_{j\neq i,k}^{N} w_j^i Y_{jt} - w_k^i \hat{Y}_{kt}\right)\right] = 0. \qquad (17)$$

For units in which **A2**(a) does not hold, $a_i(\boldsymbol{w}) = 0$.

Note that the parameter set $\mathcal{W}_i$ (for all i) is compact by definition. By condition **A1**, $a_i(\boldsymbol{\phi})M_i(\boldsymbol{\phi})$ is bounded and continuous for each $\boldsymbol{\phi}$ with probability 1. Given these properties and **A4**, we have uniform convergence of the moment conditions by Theorem 2.3 in White and Domowitz (1984). Thus, $\sum_{j\neq i,k}^{N} \hat{w}_j^i Y_{jt} + \hat{w}_k^i \hat{Y}_{kt} \xrightarrow{p} L_{it}$ for all (i,t). Note that the $\boldsymbol{w}$ weights are not necessarily unique, but the predicted counterfactuals are.

Moreover, for units in which **A2**(a) holds, $M_i(\hat{\boldsymbol{w}}) \xrightarrow{p} 0$ and $a_i(\hat{\boldsymbol{w}}) \xrightarrow{p} \overline{a}_i \geq 0$ where $\overline{a}_i > 0$ by condition **A5**. For units in which **A2**(a) does not hold, $M_i(\hat{\boldsymbol{w}}) \xrightarrow{p} c > 0$ such that $a_i(\hat{\boldsymbol{w}}) \xrightarrow{p} 0$. Therefore, if $\sum_{j\neq i,k}^{N} \hat{w}_j^i Y_{jt} + \hat{w}_k^i \hat{Y}_{kt} \xrightarrow{p} L_{it}$ for all (i,t), then $a_i(\hat{\boldsymbol{w}})$ converges to weights meeting the conditions of equation (7).

Part 2: Part 1 assumed given a_i weights for equation (13). Given a_i without these same properties, equation (17) is not equal to 0. The objective function in minimized at $a_i(\boldsymbol{w})$ and $\boldsymbol{w}$. Given the properties discussed above, this implies both $\sum_{j\neq i,k}^{N} \hat{w}_j^{i,(S-1)} Y_{jt} + \hat{w}_k^{i,(S-1)} \hat{Y}_{kt} \xrightarrow{p}$

L_{it} and $\sum_{j \neq i,k}^{N} \hat{w}_j^{i,(S)} Y_{jt} + \hat{w}_k^{i,(S)} \hat{Y}_{kt} \xrightarrow{p} L_{it}$, where $\hat{\boldsymbol{w}} \equiv \hat{\boldsymbol{w}}^{(S)}$.

Part 3: I now consider the estimate of the treatment effect. For a unit i in which **A2**(a) holds, then $a_i(\hat{\boldsymbol{w}}) \xrightarrow{p} \overline{a}_i > 0$. In all other cases, $a_i(\hat{\boldsymbol{w}}) \xrightarrow{p} 0$ so these units can be ignored (asymptotically).

Consider unit 1 for $t > T_0$ assuming that **A2**(a) holds. By consistency of $\sum_{j \neq i}^{N} \hat{w}_j^i L_{jt}$,

$$\hat{\alpha}_{1t} \xrightarrow{p} \alpha_{1t} + \left(L_{1t} - \sum_{j=2}^{N} w_j^1 L_{jt} \right) + \left(\epsilon_{1t} - \sum_{j=2}^{N} w_j^1 \epsilon_{jt} \right).$$

We know that

$$\begin{aligned} E\left[L_{1t} - \sum_{j=2}^{N} w_j^1 L_{jt} \right] &= 0, \\ \text{and } E\left[\epsilon_{1t} - \sum_{j=2}^{N} w_j^1 \epsilon_{jt} \right] &= 0. \end{aligned}$$

Alternatively, consider unit i in which **A2**(b) holds.

$$Y_{it} - \sum_{j \neq i} \hat{w}_j^i Y_{jt} = -\hat{\alpha}_{1t} \hat{w}_1^i + \left(L_{it} - \sum_{j \neq i} \hat{w}_j^i L_{jt} \right) + \left(\epsilon_{it} - \sum_{j \neq i} \hat{w}_j^i \epsilon_{jt} \right)$$

The last two terms converge to quantities with expectation zero, as before. Thus, $\hat{\alpha}_{1t}$ is asymptotically unbiased.

□
